# A Case of Metastatic Pulmonary Calcification

**DOI:** 10.5334/jbsr.4210

**Published:** 2026-03-03

**Authors:** Diane Lefebvre, Pascale Bohy

**Affiliations:** 1 Hôpital Erasme - Route de Lennik, 808, 1070 Bruxelles, Belgium

**Keywords:** chest, CT, pulmonary calcifications, metabolic disorders

## Abstract

Pulmonary calcifications are a frequent phenomenon with a wide range of etiology. They are divided into two categories: metastatic and dystrophic pulmonary calcification, with different underlying pathology and CT features.

*Teaching point:* Metastatic pulmonary calcifications appear when there is a metabolic imbalance with some typical radiographic features for characterization.

## Introduction

Pulmonary calcifications are a frequent phenomenon and are divided into two categories: metastatic pulmonary calcification (MPC) and dystrophic pulmonary calcification (DPC). These categories differ by their etiology: MPC is caused by systemic hypercalcemia and DPC by local injuries [1]. The CT features are slightly different: MPC more often shows diffuse calcified nodules, which can be amorphous and associated with sand‑like, fine ground‑glass opacities that can also evolve to consolidative opacities. DPC appears to be more nodular lesions and is likely to be associated with lung damage, such as infection or fibrosis [2]. Pulmonary calcification may also occur in association with protein deposition disease, such as pulmonary amyloidosis, in benign neoplasms or metastatic disease. Clinical context and knowledge of underlying pathologies contribute to the assessment of pulmonary calcification [3].

## Case Report

A 43‑year‑old male patient underwent liver transplantation (2013) for hepatocellular carcinoma and cirrhosis due to HBV and HDV co‑infection. There was an immediate postoperative complication with a hemorrhagic shock due to leakage at the inferior vena cava and acute tubular necrosis with renal failure. In 2024, the patient developed acute ischemic cholangitis with encephalopathy and thrombocytopenia, ultimately requiring a new liver transplant.

**Figure 1:**
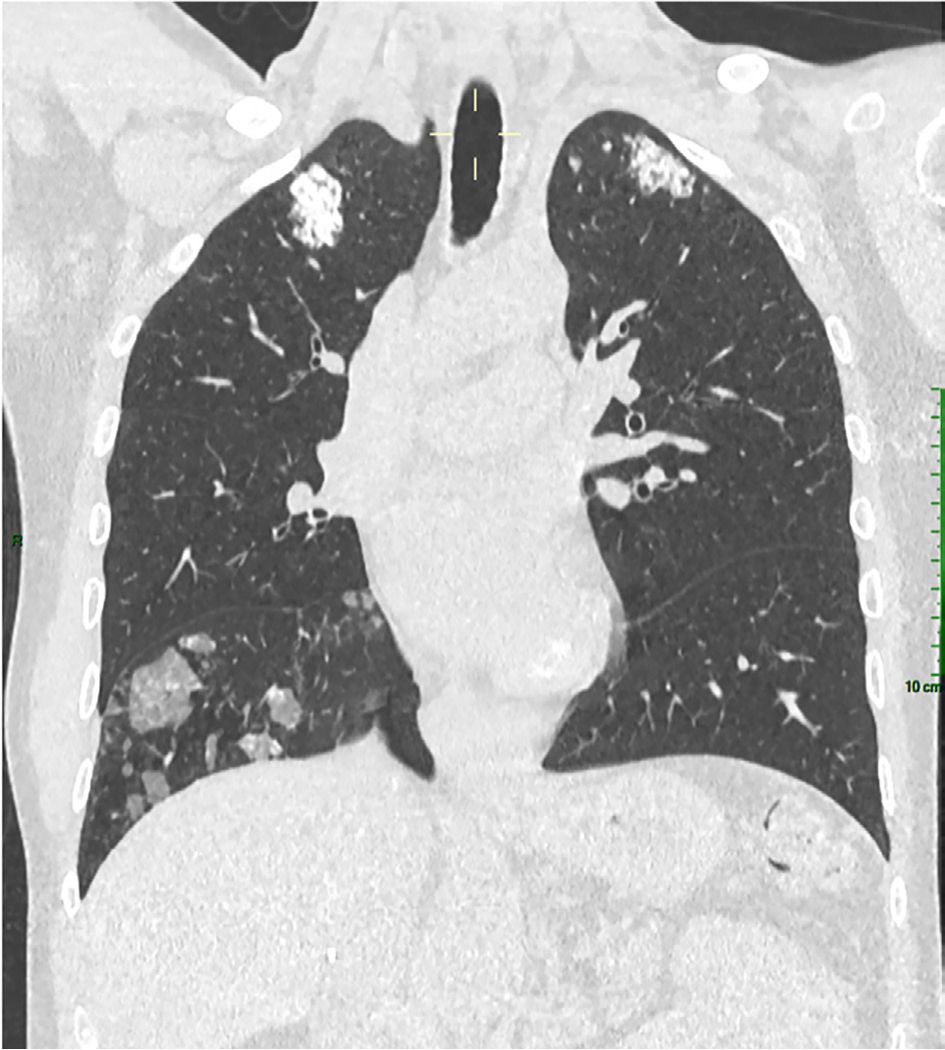
Coronal chest CT with bilateral ground‑glass nodular opacities, partially calcified, predominant in the superior regions.

**Figure 2:**
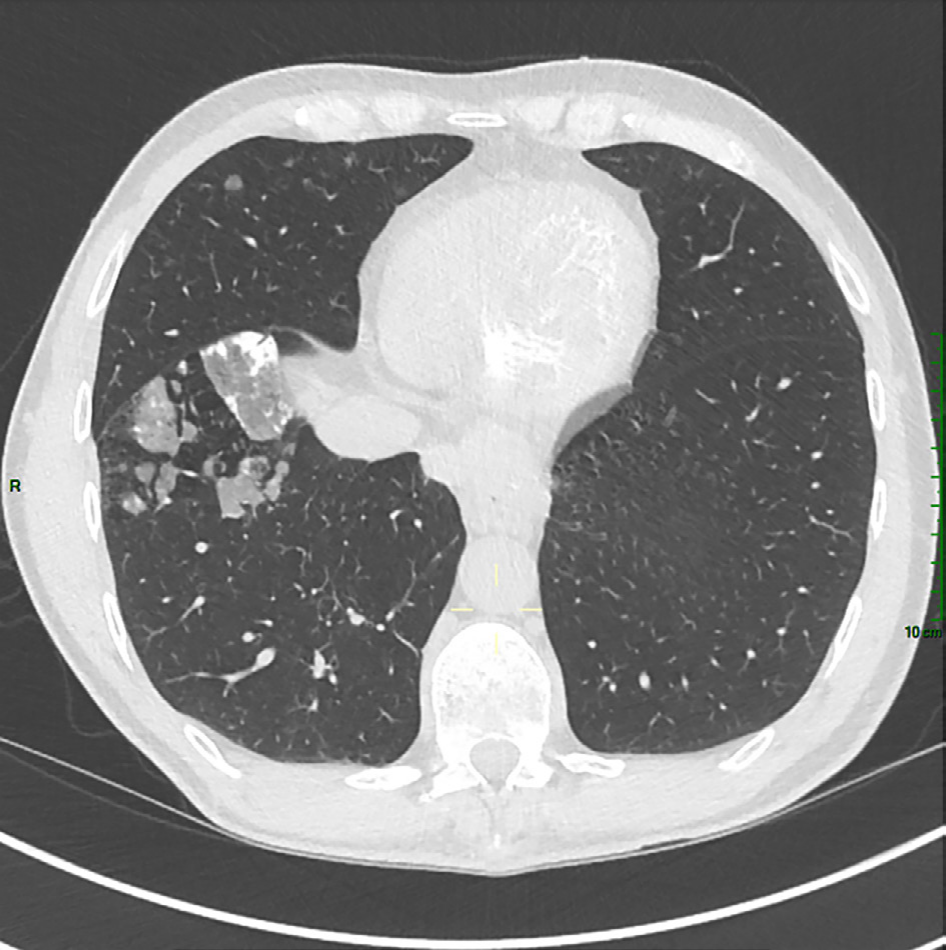
Axial chest CT: ground‑glass nodular opacities, partially calcified.

**Figure 3:**
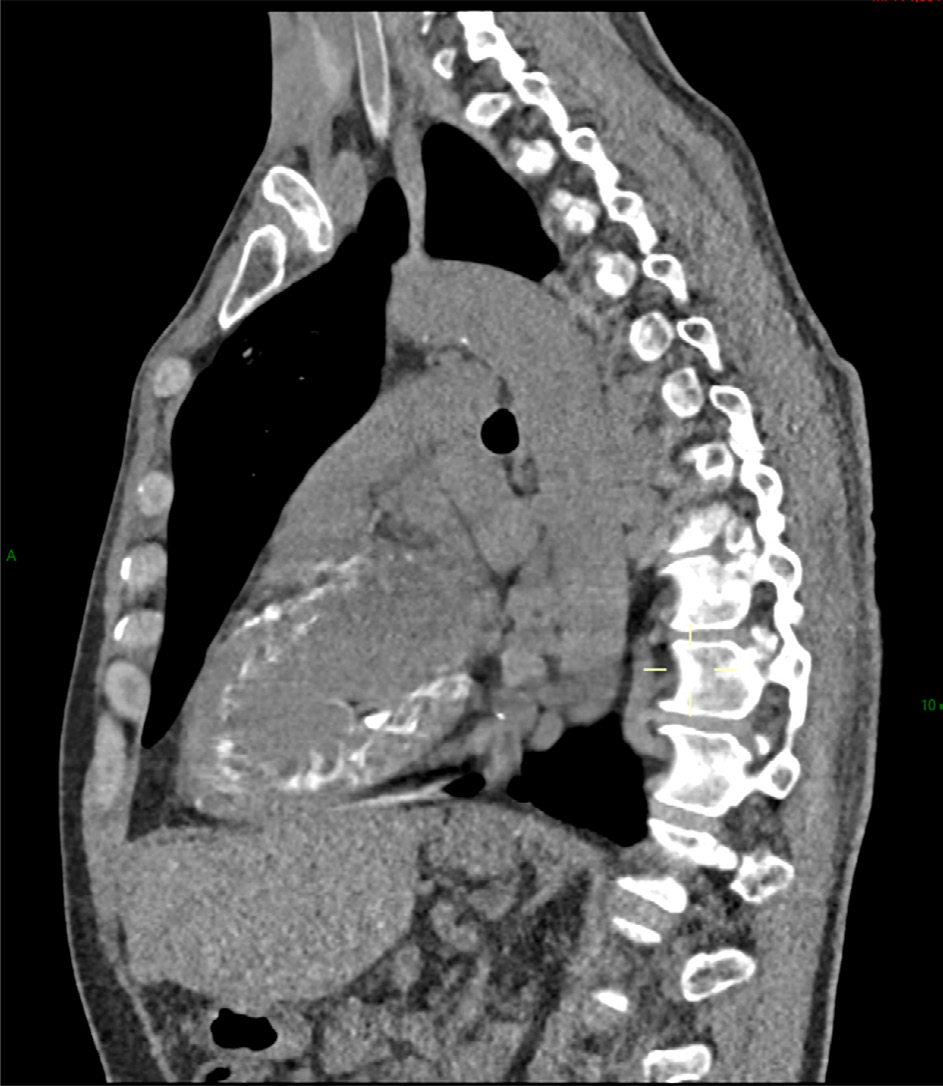
Sagittal chest CT: parietal left cardiac ventricle calcifications (porcelain heart).

In 2024, the patient underwent a chest CT during the follow‑up of hepatocellular carcinoma (Figures 1–3). Several abnormalities were observed: bilateral ground‑glass nodular opacities, partially calcified, predominant in the anterior inferior and superior regions, and parietal left cardiac ventricle calcifications (porcelain heart).

The final diagnosis was metastatic pulmonary calcifications due to hepatic transplantation and renal failure.

## Discussion

Pulmonary calcifications can be divided into two types: MPC and DPC, with different etiology, underlying physiopathology, distribution, topography, size, and the presence of ground‑glass opacities. Causes of MPC include dysmetabolism such as hypercalcemia (hyperparathyroidism, cancer, vitamin D excess, prolonged immobilization), hyperphosphatemia (CRF, terminal renal failure, excessive phosphate intake), or post‑transplantation (heart and liver). DPC can be provoked by chronic pulmonary disease (COPD, ILD), infections (tuberculosis, histoplasmosis, pneumonia), inflammatory disease (sarcoidosis, silicosis), autoimmune disease (rheumatoid arthritis), amyloidosis, and neoplastic disease (hamartoma, chondroma, neuroendocrine pulmonary tumor, metastasis).

The physiopathology of MPC relies on high serum calcium levels, and anomalies occur in a normal lung parenchyma. The lung anomalies are bilateral, rather small sized, and diffuse and symmetric with a light predominance to the superior lobes in MPC. Contrarily, serum calcium levels are normal in DPC and appear on a pathological lung parenchyma, more likely with an asymmetric distribution predominately in the pathological lung zones and with an irregular shape and variability in size.

Finally, ground‑glass opacities are constant in MPC and absent in DPC.

## Conclusion

Metastatic pulmonary calcifications appear when there is a metabolic imbalance (hypercalcemia and hyperphosphatemia) and result from an interstitial process with deposition of calcium salts in the basement membrane of the alveolar epithelium. Anomalies are most seen in upper pulmonary zones, where the ventilation–perfusion ratio is higher.

On the chest CT scan, they are characterized by multiple, diffuse calcified nodules (3–10 mm), ground‑glass opacities and diffuse consolidations, lobar atelectasis, myocardial calcifications, and calcification of the bronchial wall, of the pulmonary arterioles, and of the superior vena cava.
